# Knowledge of specific HIV transmission modes in relation to HIV infection in Mozambique

**DOI:** 10.12688/f1000research.1-1.v1

**Published:** 2012-07-13

**Authors:** Devon D Brewer

**Affiliations:** 1 Interdisciplinary Scientific Research, Seattle, WA, USA

## Abstract

**Background:** In prior research, Africans who knew about blood-borne risks were modestly less likely to be HIV-infected than those who were not aware of such risks.

**Objectives/Methods:** I examined the association between knowledge of specific HIV transmission modes and prevalent HIV infection with data from the 2009 Mozambique AIDS Indicator Survey.

**Results:** Respondents displayed high awareness of blood exposures and vaginal sex as modes of HIV transmission. However, only about half of respondents were aware of anal sex as a way HIV can be transmitted. After adjustments for demographics and sexual behaviors, respondents who knew that HIV could spread by contact with infected blood or by sharing injection needles or razor blades were less likely to be infected than those who did not know about these risks. Respondents who knew about sexual risks were as, or more, likely to be HIV infected as those who did not know about sexual risks. Also, children of HIV-uninfected mothers were less likely to be infected if their mothers were aware of blood-borne HIV risks than if their mothers were unaware.

**Conclusion:** HIV education campaigns in Mozambique and elsewhere in sub-Saharan Africa should include a focus on risks from blood exposures and anal sex.

## Introduction

A wide variety of blood exposures is independently associated with incident and prevalent HIV infection in sub-Saharan Africa ^1– 9^. Anal sex has also been repeatedly associated with HIV infection in heterosexual Africans ^10– 15^, with some exceptions ^16, 17^. Knowledge of blood-borne HIV risks varies markedly across the region, with awareness much higher in West, central and East Africa than in southern Africa ^18^. This geographic variation in knowledge corresponds closely to differences in HIV prevalence, such that countries in which many people know about blood-borne risks have much lower HIV prevalence than countries in which few know about these risks. Although HIV education campaigns in West, central and East Africa have included a focus on blood-borne risks, campaigns in Southern Africa have not, likely leading to deficits in Southern Africans’ knowledge. At the individual level, persons who know about blood-borne risks are modestly less likely to be HIV-infected than those who are not aware of such risks, independent of demographics and sexual behaviors ^18^.

In contrast, belief in condom use as a strategy for avoiding HIV infection is positively associated with HIV infection at both the individual and national levels in sub-Saharan Africa ^18^. That is, those who believe condoms are an effective strategy for preventing HIV transmission are more likely to be HIV-infected than those who do not believe condom use protects against HIV, independent of demographics and sexual behaviors.

Although long assumed to be rare, anal sex is not uncommon in sub-Saharan Africa, as modest to large proportions of heterosexual men and women report engaging in it ^10– 15, 19– 27^. Research based on nonprobability samples suggests that many Africans are unaware that anal sex is a significant risk for HIV transmission. While 87% of young adult Nigerians in one study knew anal sex is an HIV risk ^28^, only 54% of men in Cape Town, South Africa in 1999 ^19^ and 29% of adolescents in a Western Cape (South Africa) township with very high HIV prevalence shared that perception ^26^. Some South African women in a qualitative study explained the lack of risk involved with anal sex ^29^. Moreover, Kenyan prostitutes viewed vaginal sex as equally or more risky than anal sex ^21^. Throughout the last 13 years, researchers have noted that HIV education campaigns targeting heterosexuals in sub-Saharan Africa lack warnings about the risk of anal sex ^10, 15, 19, 23, 24, 27, 30^.

In this paper, I report on the relationship between knowledge of HIV transmission routes and prevalent HIV infection in Mozambique, a country with high HIV prevalence not included in prior analyses. I analyzed data from the 2009 Mozamibique AIDS Indicator Survey (AIS), a special version of the Demographic and Health Surveys (DHS) conducted in poor countries. The 2009 Mozambique AIS differs from prior DHS with respect to how knowledge of HIV transmission risks was measured. In the 2009 Mozambique AIS respondents were asked directly about several specific blood-borne and sexual transmission routes. The 2009 Mozambique AIS also involved HIV testing of children, enabling examination of the association between mothers’ knowledge of blood-borne HIV transmission routes and HIV infection in their children.

## Methods

### 2009 Mozambique AIDS Indicator Survey (AIS) data

In the 2009 Mozambique AIS, the household participation rate was 99%, and 95% of eligible women (age 15-64) and 90% of eligible men (age 15–64) participated ^31^. Ninety-two percent of adult respondents and 87% of children between ages 0 and 11 years provided dried blood spot specimens for HIV testing. HIV serostatus was determined with an enzyme-linked immunosorbent assay (ELISA) test and confirmed by a different ELISA test. Specimens with discrepant ELISA results were confirmed by a further ELISA test. The 2009 Mozambique AIS data and documentation are available at http://www.measuredhs.com.

I included respondents’ data in analysis if they reported never having been tested previously for HIV and also reported awareness of HIV/AIDS. HIV counseling may, in some places, involve education about blood-borne and sexual risks ^32^, which may confound the relationship between knowledge and serostatus ^33^. Respondents who did not report awareness of HIV/AIDS were not asked about ways that it could be transmitted. Ninety-seven percent of female respondents and 99% of male respondents reported they had heard of HIV/AIDS.

Respondents who reported awareness of HIV/AIDS were asked whether it was possible to transmit HIV through each of several types of direct and indirect interpersonal contact. I focused on knowledge of four types of contact: sharing injection needles/blades; contact with infected blood; unprotected vaginal sex (i.e., vaginal sex without a condom); and unprotected anal sex. I coded "don’t know" responses to these questions as "no".

For the analyses of mother’s knowledge and child’s serostatus, I included biological mother-child pairs in which the mother was HIV-uninfected, to eliminate confounding by vertical transmission.

### 2003 Mozambique Demographic and Health Survey (DHS) data

In the 2003 Mozambique DHS, the household participation rate was 95%, and 91% of eligible women (age 15-49 years) and 81% of eligible men (age 15–64 years) participated ^34^. Women were recruited as respondents from all households, but men were recruited as respondents only from one-third of households. Respondents did not provide blood specimens for HIV testing. The 2003 Mozambique DHS data and documentation are also available at http://www.measuredhs.com.

As I did with the AIS data, I included respondents’ data in analysis if they reported never having been tested previously for HIV and also reported awareness of HIV/AIDS. Ninety-seven percent of female respondents and 99% of male respondents in the 2003 DHS reported they had heard of HIV/AIDS.

Respondents who reported awareness of HIV/AIDS were asked "Do you know how you can avoid HIV/AIDS?" Those answering affirmatively were then asked "How can you prevent HIV/AIDS?" Interviewers encouraged and recorded multiple responses, and responses were coded by DHS staff. To compare with the 2009 Mozambique AIS and other DHS, I focused on two measures: whether a respondent had a response that was coded as "only use sterilized syringes/needles" and "always use a condom". I coded "don’t know" responses to the open-ended question as "no" for each of these measures.

### Statistical analysis

With the 2009 Mozambique AIS data, I computed cross-classifications, odds ratios, and the associated 95% confidence intervals (CI) for the relationships between each knowledge measure and prevalent HIV infection, separately for women and men. To examine the associations further, I calculated the odds ratios, with HIV status as the dependent variable and a knowledge measure as the independent variable, adjusted for age (in whole years), urban/rural residence, number of whole years of education, wealth (in quintiles), reported number of sex partners in the prior 12 months, and whether the respondent reported having had a sexually transmitted disease in the prior 12 months. In addition, I calculated adjusted odds ratios (AORs) in the manner just described but adjusting for each of the three other knowledge measures as well. I also computed the Pearson (phi) correlation between each pair of knowledge measures. I used SPSS 7.5 (SPSS Inc., Chicago, USA) to perform the analyses. I compared the adjusted odds ratios for Mozambique with similar DHS results from four countries in Southern Africa (Lesotho, Malawi, Swaziland, and Zimbabwe) based on knowledge measures derived from free responses to an open-ended question about ways to avoid HIV infection ^18^.

### Online search of HIV prevention education efforts focused on blood-borne risks

In July of 2011, I sought evidence of public HIV education campaigns focused on blood-borne risks in Mozambique by searching two online databases: the Google search engine and the Media/Materials Clearinghouse. For the Google search, I used the key words "Mozambique", "HIV", and "razor". I examined the resources identified until I found 30 consecutive resources to be irrelevant (for a total 320 resources examined). I repeated this search in September of 2011 at the Google’s Mozambican site, with the equivalent key words in Portuguese: "Moçambique", "VIH," and "lâmina" (290 resources examined). I also extended this search by adding, in turn, the names of the 14 HIV prevention programs and campaigns mentioned in the AIS questionnaire (for questions about respondents’ familiarity with the programs and campaigns) ^31^. My Google search was not exhaustive, especially given the limited scope induced by the key word "razor". However, the razor blade is perhaps the most ubiquitous sharp instrument involving possible blood exposure in daily life in Mozambique. Any public education campaign about blood-borne HIV risks that excludes explicit mention of razor blades is probably superficial or very narrow. For the Media/Materials Clearinghouse search, I inspected all materials pertaining to Mozambique. I also examined any sources related to Mozambique that I had found in an earlier search of the US National Library of Medicine Gateway ^18^.

## Results

In the 2009 Mozambique AIS, over 90% of respondents reported that HIV could spread through shared needles/razors and nearly 80% reported that HIV could be spread through contact with infected blood ( Table 1). Approximately 90% of women and men reported vaginal sex as an HIV transmission route. In contrast, only 48% of women and 58% of men thought that HIV could spread through anal sex.

**Table 1.** Knowledge of specific HIV transmission modes and relationship with prevalent HIV infection, Mozambique, 2009.

	Women				Men			
			HIV prevalence				HIV prevalence	
			acknowledged?				acknowledged?	
Knowledge measure	N	% acknowledging	Yes	No	N	% acknowledging	Yes	No
Needles/razors	3383	90.4	12.2	12.0	3391	92.6	8.5	7.6
Blood contact	3378	77.3	11.7	13.8	3390	78.7	8.6	7.8
Vaginal sex	3381	87.1	12.5	10.3	3385	91.9	8.8	4.4
Anal sex	3378	47.9	13.6	10.9	3383	58.2	8.9	7.7

Before adjustments for demographics and sexual behavior variables, respondents who believed that HIV spread through vaginal or anal sex were more likely to be infected than those who did not believe HIV spread in that way ( Table 1). In unadjusted analyses, respondents who reported that HIV could be transmitted through blood contact or reusing sharps were about as likely to be infected as respondents who did not think HIV was transmitted in that way.

Urban residence, education, and wealth were moderately positively associated with knowledge of blood-borne HIV risks (results not shown), and these same factors were positively related to HIV infection as well ^31^. Consequently, the AORs between the knowledge measures and HIV infection in ( Table 2 and Table 3) provide better estimates of the relationship between the knowledge measures and HIV infection.

**Table 2.** Associations between measures of knowledge of HIV transmission modes and prevalent HIV infection, Mozambican women, 2009.

		AOR single measure ^1^		
Knowledge measure	Bivariate OR	Mozambique	4 Southern African countries ^2^	AOR all measures ^3^
Needles/razors	1.02 (0.72–1.45)	0.89 (0.62–1.27)	0.80 (0.70–0.92)	0.93 (0.63–1.37)
Blood contact	0.83 (0.65–1.05)	0.81 (0.63–1.03)	—	0.80 (0.62–1.03)
Vaginal sex	1.24 (0.89–1.72)	1.06 (0.76–1.49)	1.26 (1.13–1.42)	1.04 (0.72–1.49)
Anal sex	1.28 (1.04–1.58)	1.18 (0.95–1.46)	—	1.21 (0.96–1.51)

Table 2 Note: 95% confidence intervals in parentheses

^1^Adjusted for age, urban/rural residence, education, wealth, reported number of sex partners in the prior 12 months, and reported sexually transmitted disease in the prior 12 months

^2^Summary of results from Lesotho, Malawi, Swaziland, and Zimbabwe for similar knowledge measure

^3^Includes same adjustments as for single measure AOR, but also adjusted for the 3 other knowledge measures

**Table 3.** Associations between measures of knowledge of HIV transmission modes and prevalent HIV infection, Mozambican men, 2009.

		AOR single measure ^1^		
Knowledge measure	Bivariate OR	Mozambique	4 Southern African countries ^2^	AOR all measures ^3^
Needles/razors	1.13 (0.70–1.83)	0.91 (0.55–1.49)	0.86 (0.71–1.04)	0.87 (0.52–1.45)
Blood contact	1.11 (0.82–1.51)	0.95 (0.70–1.30)	—	0.93 (0.67–1.30)
Vaginal sex	2.10 (1.16–3.80)	1.85 (0.99–3.45)	1.21 (1.07–1.37)	1.93 (1.02–3.64)
Anal sex	1.18 (0.92–1.51)	1.03 (0.79–1.34)	—	0.98 (0.75–1.28)

Table 3 Note: 95% confidence intervals in parentheses

^1^Adjusted for age, urban/rural residence, education, wealth, reported number of sex partners in the prior 12 months, and reported sexually transmitted disease in the prior 12 months

^2^Summary of results from Lesotho, Malawi, Swaziland, and Zimbabwe for similar knowledge measure

^3^Includes same adjustments as for single measure AOR, but also adjusted for the 3 other knowledge measures

After adjustments for demographic factors and sexual behavior variables, the measures of knowledge of blood-borne risks become inversely related to HIV infection. That is, respondents who reported that HIV can spread through shared needles/razors and blood contact were less likely to be infected than those who reported that HIV does not spread through such means, once demographics and sexual behaviors were held constant. The adjustments for potential confounders also caused the positive associations for knowledge of vaginal sex risk in women and knowledge of anal sex risk in men to disappear. However, the adjusted associations for knowledge of anal sex risk in women and knowledge of vaginal sex risk in men remained meaningfully positive. In particular, men who believed HIV spreads through vaginal sex were almost twice as likely to be infected as men who did not believe this ( Table 3).

The adjusted odds ratios for knowledge of shared needles/razors as transmission risks are generally similar to those for knowledge of avoiding shared razors as an HIV prevention strategy in four other countries in Southern Africa ( Table 2 and Table 3). The adjusted associations for knowledge of vaginal sex risk also approximate those for knowledge of condoms as a prevention strategy from the other Southern African countries.

The blood-borne risk knowledge measures correlated moderately with each other (r = 0.26–0.29) as did the sexual risk knowledge measures (r = 0.23–0.29). The associations between one blood-borne risk knowledge measure and one sexual risk knowledge measure were weaker (r = 0.12–0.23). Nonetheless, when all four knowledge measures were included in analysis together (last column in ( Table 2 and Table 3), the associations between each knowledge measure and HIV infection were almost the same as when the knowledge measures were analyzed separately.

HIV uninfected mothers’ knowledge of blood-borne HIV risks was also inversely related to their children’s HIV status. Children whose mothers knew that contact with blood is an HIV risk were less likely to be infected (0.4%; 11/2511) than children whose mothers did not know about this risk (1.2%; 7/591; OR 0.37, 95% CI 0.14–0.95). Similarly, children whose mothers knew that sharing needles and razors was a risk were also less likely to be infected (0.5%, 16/2918) than children whose mothers were unaware of this risk (1.1%, 2/185; OR 0.50, 95% CI 0.12–2.21).

In the 2003 Mozambique DHS, 9.8% (1110/11381) of women and 18.9% (413/2709) of men recalled "only use sterilized syringes/needles" as a way to prevent HIV. However, 55% (6253/11381) of women and 68% (1845/2709) of men recalled "always use a condom" as a way to prevent HIV.

There was no online evidence of public HIV education campaigns that focused on blood-borne transmission risks in Mozambique. Some participants in a face-to-face community HIV education program that was implemented in Mozambique credited the program with informing them about some blood-borne risks ^35, 36^. However, none of the descriptions of the program at the program’s website ( http://www.steppingstonesfeedback.org) or elsewhere online indicate that this topic is formally covered in the program.

## Discussion

Adult Mozambicans displayed very high awareness of some blood exposures and vaginal sex as modes of HIV transmission when directly asked about such contact in the 2009 Mozambique AIDS Indicator Survey. However, only a little more than half of respondents were aware of anal sex as a way HIV can be transmitted. After adjustments for demographics and sexual behaviors, knowledge of blood-borne risks was inversely associated with prevalent HIV infection. That is, respondents who knew that HIV could spread by contact with infected blood or by sharing injection needles or razor blades were less likely to be infected than those who did not know about these risks. Knowledge of sexual risks was negligibly to positively related to prevalent HIV infection. The positive association was strongest in men: those acknowledging unprotected vaginal sex as a risk were almost twice as likely to be infected with HIV as those not acknowledging. These associations between knowledge of blood-borne and sexual risks and prevalent HIV infection in Mozambique are similar in magnitude to those observed in four other Southern African countries based on roughly parallel knowledge measures. In addition, mothers’ knowledge of blood-borne risks was inversely associated with HIV infection in their children, which suggests that persons aware of such risks not only are more likely to take corresponding preventive measures for themselves but for those in their care as well. Moreover, there was no online evidence that public HIV education campaigns in Mozambique have included a meaningful focus on blood-borne risks.

The very high proportion of Mozambicans who were aware of blood-borne HIV risks (approximately 90%) in the 2009 AIS contrasts sharply with the very low proportions of 2003 Mozambique DHS respondents and DHS respondents in four other Southern African countries who mentioned such risks (8–23%) ^18^. This difference seems to be due to assessing knowledge in the 2009 Mozambique AIS by *recognition* of specific risks and in the other DHS by *recall* of risks. In South Africa and Swaziland, the discrepancies between levels of knowledge of blood-borne HIV risks when measured by recognition ^18, 26, 37^ and recall ^18, 38– 42^ are even larger. These combined results refute my earlier hypothesis ^18^ that Southern Africans and perhaps Africans generally know that contaminated blood can transmit HIV only when the exposure is visible and large. Instead, these results indicate that Southern Africans are typically aware of a variety of blood-borne HIV risks (including shared needles and razors that might not involve large or visible blood contamination), but only when specifically prompted.

Interestingly, observed levels of Southern Africans’ knowledge of sexual risks differ relatively little based on measurement approach. In the aforementioned studies, the proportions of respondents aware of sexual HIV transmission risks tended to be only slightly less when measured with recall methods than recognition methods. The difference between the proportion of 2003 Mozambique DHS respondents who recalled "always use a condom" as a prevention strategy (62%) and the proportion of 2009 Mozambique AIS respondents who recognized vaginal sex as a transmission mode (92%) is somewhat larger, although this could be due to increases over time in Mozambicans’ perceptions of vaginal sex as a risk.

The discrepancy between observed levels of knowledge of blood-borne HIV risks based on recall and recognition measurement reveals that blood-borne risks are not salient in the minds of Southern Africans. In 2011, Class interviewed 23 HIV infected adults and 26 parents or other caretakers of HIV infected children in Maputo, Mozambique (Deena Class, personal communications, July 11, 2011, August 27, 2011, and September 29, 2011). Nearly all of Class’ respondents noted blood-borne transmission as one mode of HIV spread. However, none of the adults attributed their infections to a blood exposure. Five of the infected children had seronegative biological mothers, indicating that these children had acquired their infections horizontally. Remarkably, only one of these mothers suspected blood-borne transmission for her child. Furthermore, none of these five mothers were told by their healthcare providers how their children might have been infected. In another study, Zimbabwean youth almost universally recognized the potential for HIV transmission through shared razors, yet ranked the avoidance of sharing razors as the least effective strategy for preventing HIV in comparison to strategies focused on sexual exposures and behaviors not known to protect against infection (avoiding deep kissing and washing hands) ^43^. Similarly, Kenyans in Nyanza province grossly underestimated how long HIV can survive on a blood-contaminated razor blade or inside a blood-contaminated needle or syringe ^44^. Thus, many Africans may recognize blood-borne HIV transmission as a theoretical possibility, but regard it as unlikely in practice.

Such apparent misunderstandings of blood-borne HIV transmission are not surprising given the lack of public education about blood-borne risks in sub-Saharan Africa. Just as in other African countries ^18^, efforts to educate Mozambican traditional healers about hygienic practices began decades ago ^45, 46^ and continue to this day ^47, 48^. There was also at least one attempt to educate barbers about blood-borne risks in the early 1990s ^49^. However, I found no good evidence that public HIV education campaigns in Mozambique have in the past focused on blood-borne risks to any meaningful extent. Furthermore, as in other Southern African countries ^18^, large recent and current public HIV education campaigns in Mozambique exclude mention of blood-borne risks ^50, 51^ (see also: One Love Regional Campaign – Southern Africa, and Center for Communication Programs). Indeed, Class’ Mozambican respondents who noted blood-borne transmission as one mode of HIV spread said they learned of these risks informally, not from HIV education campaigns, health care providers, or public health sources (Deena Class, personal communication, September 29, 2011).

Mozambicans’ awareness of anal sex as a transmission risk was much lower than their awareness of vaginal sex as a risk, even though the actual risk from anal sex is much higher. Contrary to my expectation, knowledge of anal sex risk was not associated with lower rate of HIV infection. It is unclear why this is so. Further investigation of the perceived risk of anal sex (e.g., whether risk differs for men and women) and whether respondents engage in anal sex might help clarify this result.

Beliefs in vaginal sex as an HIV transmission risk and condom use as an HIV prevention strategy ^18^ are associated with a higher likelihood of HIV infection in Mozambique and elsewhere in sub-Saharan Africa. This is consistent with the lack of association between condom use in vaginal sex and incident HIV infection ^14, 52, 53^. Condom use itself may even make people more vulnerable to infection by reducing mucosal exposure to HIV and thus hindering the development of alloimmunity against HIV ^54, 55^.

To determine the modes of transmission of HIV in sub-Saharan Africa with confidence, researchers must assess blood and sexual exposures comprehensively in incident HIV cases and controls, trace their contacts corresponding to such exposures, and sequence the DNA in infected persons’ HIV isolates ^56– 58^. In the meantime, HIV education campaigns in Mozambique and other poor countries should focus on blood-borne and anal sex transmission risks comprehensively and emphasize strategies for avoiding these dangers ^59– 61^.

## References

[1] St Lawrence JS Klaskala W Kankasa C: Factors associated with HIV prevalence in a pre-partum cohort of Zambian women. *Int J STD AIDS.* 2006;17(9):607–613 10.1258/095646206778113050 16942652

[2] Deuchert E: Maternal health care and the spread of AIDS in Burkina Faso and Cameroon. *World Health Pop.* 2007;9(3):55–72 10.12927/whp.2007.19344 18272943

[3] Deuchert E Brody S: The role of health care in the spread of HIV/AIDS in sub-Saharan Africa: evidence from Kenya. *Int J STD AIDS.* 2006;17(11):749–752 10.1258/095646206778691167 17062178

[4] Brewer DD Potterat JJ Roberts JM Jr: Male and female circumcision associated with prevalent HIV infection in virgins and adolescents in Kenya, Lesotho, and Tanzania. *Ann Epidemiol.* 2007;17(3):217–226 10.1016/j.annepidem.2006.10.010 17320788

[5] Gisselquist D: Points to consider: Responses to HIV/AIDS in Africa, Asia, and the Caribbean.Adonis and Abbey Publishers Ltd, London;2007 Reference Source

[6] Peters EJ Brewer DD Udonwa NE: Diverse blood exposures associated with incident HIV infection in Calabar, Nigeria. *Int J STD AIDS.* 2009;20(12):846–851 10.1258/ijsa.2009.009272 19948899

[7] Okinyi M Brewer DD Potterat JJ: Horizontally acquired HIV infection in Kenyan and Swazi children. *Int J STD AIDS.* 2009;20(12):852–857 10.1258/ijsa.2009.009204 19948900

[8] Vaz P Pedro A Le Bozec S: Nonvertical, nonsexual transmission of human immunodeficiency virus in children. *Pediatr Infect Dis J.* 2010;29(3):271–274 10.1097/INF.0b013e3181c17a58 20016396

[9] Brewer DD: Scarification and male circumcision associated with HIV infection in Mozambican children and youth. *WebmedCentral EPIDEMIOLOGY* 2011,2(9):WMC002206 Reference Source

[10] Lane T Pettifor A Pascoe S: Heterosexual anal intercourse increases risk of HIV infection among young South African men. *AIDS.* 2006;20(1):123–125 10.1097/01.aids.0000198083.55078.02 16327330

[11] Mlotshwa M Abdool Karim Q Frohlich J: Risk factors for HIV infection in young women in rural South Africa: implications for HIV prevention trials.Abstract MOPE0276, 17th International AIDS Conference, Mexico City, August,2008 Reference Source

[12] Grijsen ML Graham SM Mwangome M: Screening for genital and anorectal sexually transmitted infections in HIV prevention trials in Africa. *Sex Transm Infect.* 2008;84(5):364–370 10.1136/sti.2007.028852 18375645 PMC3895478

[13] Adoga MP Banwat EB Forbi JC: Human immunodeficiency virus, hepatitis B virus and hepatitis C virus: sero-prevalence, co-infection and risk factors among prison inmates in Nasarawa State, Nigeria. *J Infect Dev Ctries.* 2009;3(7):539–547 10.3855/jidc.472 19762972

[14] Feldblum PJ Lie C Weaver MA: Baseline factors associated with incident HIV and STI in four microbicide trials. *Sex Transm Dis.* 2010;37(10):594–601 10.1097/OLQ.0b013e3181e15f0b 20879087

[15] Kalichman SC Simbayi LC Cain D: Heterosexual anal intercourse among community and clinical settings in Cape Town, South Africa. *Sex Transm Infect.* 2009;85(6):411–415 10.1136/sti.2008.035287 19429569 PMC3017216

[16] Mwakagile D Mmari E Makwaya C: Sexual behaviour among youths at high risk for HIV-1 infection in Dar es Salaam, Tanzania. *Sex Transm Infect.* 2001;77(4):255–259 10.1136/sti.77.4.255 11463924 PMC1744337

[17] Priddy FH Wakasiaka S Hoang TD: Anal sex, vaginal practices, and HIV incidence in female sex workers in urban Kenya: implications for the development of intravaginal HIV prevention methods. *AIDS Res Hum Retroviruses.* 2011;27(10):1067–72 10.1089/aid.2010.0362 21406032 PMC3186689

[18] Brewer DD: Knowledge of blood-borne transmission risk is inversely associated with HIV infection in sub-Saharan Africa. *J Infect Dev Ctries.* 2011;5(3):182–198 10.3855/jidc.1308 21444987

[19] Ramjee G Gouws E Andrews A: The acceptability of a vaginal microbicide among South African men. *Int Fam Planning Perspect.* 2001;27(4):164–170 Reference Source

[20] Brody S Potterat JJ: Assessing the role of anal intercourse in the epidemiology of AIDS in Africa. *Int J STD AIDS.* 2003;14(7):431–436 10.1258/095646203322025704 12869220

[21] Ferguson A Morris C: Assessing the role of anal intercourse in the epidemiology of AIDS in Africa. *Int J STD AIDS.* 2003;14(12):856 10.1258/095646203322556228 14678597

[22] Simbayi LC Kalichman SC Jooste S: Risk factors for HIV-AIDS among youth in Cape Town, South Africa. *AIDS Behav.* 2005;9(1):53–61 10.1007/s10461-005-1681-4 15812613

[23] Schwandt M Morris C Ferguson A: Anal and dry sex in commercial sex work, and relation to risk for sexually transmitted infections and HIV in Meru, Kenya. *Sex Transm Infect.* 2006;82(5):392–396 10.1136/sti.2006.019794 16790563 PMC2563859

[24] Genberg BL Kulich M Kawichai S: HIV risk behaviors in sub-Saharan Africa and Northern Thailand: baseline behavioral data from Project Accept. *J Acquir Immune Defic Syndr.* 2008;49(3):309–319 10.1097/QAI.0b013e3181893ed0 18845954 PMC2643066

[25] Morhason-Bello IO Oladokun A Enakpene CA: Sexual behaviour of in-school adolescents in Ibadan, South-West Nigeria. *Afr J Reprod Health.* 2008;12(2):89–97 20695044

[26] DeJanes W: Knowledge, substance use, and gender differences associated with HIV infection risk among youth in a South African township. *Med Sociol Online.* 2009;4 (2):3–25 Reference Source

[27] Kazaura MR Masatu MC: Sexual practices among unmarried adolescents in Tanzania. *BMC Public Health.* 2009;9:373 10.1186/1471-2458-9-373 19804651 PMC2765439

[28] Ola TM Oludare BA: Sexual practices and knowledge about HIV/AIDS among Nigerian secondary school students. *Int J Health Res.* 2008;1:197–205 Reference Source

[29] Stadler JJ Delany S Mntambo M: Sexual coercion and sexual desire: ambivalent meanings of heterosexual anal sex in Soweto, South Africa. *AIDS Care.* 2007;19(10):1189–1193 10.1080/09540120701408134 18071961

[30] Karim SS Ramjee G: Anal sex and HIV transmission in women. *Am J Public Health.* 1998;88(8):1265–1266 10.2105/AJPH.88.8.1265-a 9702169 PMC1508299

[31] Instituto Nacional de Saúde (INS), Instituto Nacional de Estatística (INE), ICF Macro: Inquérito Nacional de Prevalência, Riscos Comportamentais e Informação sobre o HIV e SIDA em Moçambique2009. INS, INE, and ICF Macro, Calverton, Maryland, USA2010 Reference Source

[32] Centers for Disease Control and Prevention: Antenatal pre-test session flipchart. 2011

[33] Matambo R Dauya E Mutswanga J: Voluntary counseling and testing by nurse counselors: what is the role of routine repeated testing after a negative result? *Clin Infect Dis.* 2006;42(4):569–571 10.1086/499954 16421803

[34] Instituto Nacional de Estatística (INE), Ministério da Saúde (MS), ORC Macro: Moçambique Inquérito Demográfico e de Saúde2003. INE, MS, and ORC Macro, Calverton, MD, USA2005

[35] Action Aid Mozambique, Save the Children UK, UNICEF: Stepping Stones Pilot Program Evaluation: Zambezia 1999 1999 Reference Source

[36] Stepping Stones Evaluation - District of Mandimba. 2002 Reference Source

[37] Department of Health (Republic of South Africa), South African Medical Research Council: South Africa Demographic and Health Survey 1998. 2002 Reference Source

[38] Northern Cape Department of Health, Academy for Educational Development/Capable Partners Programme South Africa: A rapid assessment of the extent to which knowledge, attitude and practice of maternal nutrition, infant, and young child feeding in the context of PMTCT of HIV are integrated in the community. 2008

[39] Tuli K: Ndwedwe Child Survival Project KwaZulu Natal, South Africa. 2006 Reference Source

[40] Versteeg M Couper I Mbiza E: Knowledge, attitudes and practices regarding HIV and AIDS in the Segwaelane and Damonsville communities.Madibeng Centre for Research, Brits, South Africa;2005 Reference Source

[41] Crush J Raimundo I Simelane H: Migration-induced HIV and AIDS in rural Mozambique and Swaziland.Idasa, Cape Town;2010 Reference Source

[42] Pettifor AE Rees HV Steffenson A: HIV and sexual behaviour among young South Africans: a national survey of 15-24 year olds.Reproductive Health Research Unit, University of the Witwatersrand2004 Reference Source

[43] Banana C: An investigation into the risk behaviour regarding HIV transmission among youth in Bulawayo. *PhD thesis* University of South Africa2007 Reference Source

[44] Ounga T Okinyi M Onyuro S: Knowledge of HIV survival on skin-piercing instruments among young adults in Nyanza Province, Kenya. *Int J STD AIDS.* 2009;20(2):119–122 10.1258/ijsa.2008.008007 19182059

[45] Green EC Zokwe B Dupree JD: The experience of an AIDS prevention program focused on South African traditional healers. *Soc Sci Med.* 1995;40(4):503–515 10.1016/0277-9536(94)E0105-2 7725124

[46] Swarns RL: Mozambique enlists healers in AIDS prevention.New York Times, December 6,1999 Reference Source

[47] Mozambique News Agency: HIV/AIDS – new training manual for traditional healers.January 9,2011 Reference Source

[48] Agência de Informação de Moçambique: HIV/SIDA: em preparação manual de treino para "curandeiros".January 8, 2011. Reference Source

[49] Associacao dos Barbeiros, Mozambique Ministerio de Saude, Central de Educacao em Saude Publica: O barbeiro e o SIDA, por cada corte uma lamina nova, proteja a sua vida e a dos seus clientes [poster]; 1992

[50] Hainsworth G Zilhão I Badiani R: From inception to large scale: the Geração Biz Programme in Mozambique.World Health Organization, Geneva2009 Reference Source

[51] Hattori MK Richter K Greene J: Trust, caution, and condom use with regular partners: an evaluation of the trusted partner campaign targeting youth in four countries. *Soc Marketing Q.* 2010;16(2):18–48 Reference Source

[52] Allen S Meinzen-Derr J Kautzman M: Sexual behavior of HIV discordant couples after HIV counseling and testing. *AIDS.* 2003;17(5):733–740 10.1097/01.aids.0000050867 12646797

[53] Wawer MJ Gray RH Sewankambo NK: Rates of HIV-1 transmission per coital act, by stage of HIV-1 infection, in Rakai, Uganda. *J Infect Dis.* 2005;191(9):1403–1409 10.1086/429411 15809897

[54] Kingsley C Peters B Babaahmady K: Heterosexual and homosexual partners practising unprotected sex may develop allogeneic immunity and to a lesser extent tolerance. *PLoS ONE.* 2009;4(11):e7938 10.1371/journal.pone.0007938 19956755 PMC2775923

[55] Peters B Whittall T Babaahmady K: Effect of heterosexual intercourse on mucosal alloimmunisation and resistance to HIV-1 infection. *Lancet.* 2004;363(9408):518–524 10.1016/S0140-6736(04)15538-4 14975614

[56] Brewer DD Rothenberg RB Potterat JJ: HIV epidemiology in sub-Saharan Africa: rich in conjecture, poor in data. *Int J STD AIDS.* 2004;15:63–65 10.1258/095646204322637317

[57] Brody S Potterat JJ: Establishing valid AIDS monitoring and research in countries with generalized epidemics. *Int J STD AIDS.* 2004;15(1):1–6 10.1258/095646204322637173 14769163

[58] Brewer DD Hagan H Sullivan DG: Social structural and behavioral underpinnings of hyperendemic Hepatitis C virus transmission in drug injectors. *J Infect Dis.* 2006;194(6):764–772 10.1086/505585 16941342

[59] Gisselquist D Friedman E Potterat JJ: Four policies to reduce HIV transmission through unsterile health care. *Int J STD AIDS.* 2003;14(11):717–722 10.1258/09564620360719723 14624731

[60] Correa M Gisselquist D Gore DH: Blood-borne HIV: risks and prevention.Orient Longman Private Limited, Chennai.2008 Reference Source

[61] Gisselquist D Correa M: Don’t get stuck with HIV: how to protect yourself during health care and cosmetic procedures.2011 Reference Source

