# Supplementary material for: Knowledge of specific HIV transmission modes in relation to HIV infection in Mozambique
Source: F1000Res. 2012 Jul 13;1:1. [Version 1] doi: 10.12688/f1000research.1-1.v1 (PMC3790607; doi:10.12688/f1000research.1-1.v1)
Supplement: Peer review history

# Peer review: Referee response for version 1

**Published:** 2012-08-02

**Authors:** Carlos Morel

**Affiliations:** 1 FIOCRUZ/CDTS, Oswaldo Cruz Foundation, Ministry of Health, Rio de Janeiro, Brazil

This is an interesting article. However, I am sure there are some sections where readers would like to have some more information e.g. how the adjustments for demographic and sexual behaviors were performed.

I have read this submission. I believe that I have an appropriate level of expertise to confirm that it is of an acceptable scientific standard.

# Response

**Published:** 2012-08-02

**Authors:** Devon Brewer

**Affiliations:** Interdisciplinary Scientific Research, USA

I thank Dr. Morel for his review. I computed the adjusted odds ratios with multiple logistic regression. If there are any other points that require more information, I hope reviewers and readers will highlight those sections, so I can clarify them.

# Peer review: Referee response for version 1

**Published:** 2012-07-27

**Authors:** Nigel Livesley

**Affiliations:** 1 USAID East Africa, University Research Co., LLC, Bethesda, MD 20814, USA

This paper has a number of serious flaws.

**a) The literature is quoted selectively and issues are oversimplified.**

The paper states that blood exposure is important but does not state that the preponderance of studies show that sexual exposure is by far the most common mode of HIV transmission.

For example it doesn’t reference articles such as:

-    
Unsafe injections and transmission of HIV-1 in sub-Saharan Africa.
-    
The global burden of disease attributable to contaminated injections given in health care settings.
-    
Quantifying HIV-1 transmission due to contaminated injections.

The paper seems to claim that knowledge about blood borne infections account for the regional differences in HIV prevalence. A single citation (a paper by the same author) for this claim is provided. Other possibilities are not discussed such as:

-    
Understanding the differences between contrasting HIV epidemics in east and west Africa: results from a simulation model of the Four Cities Study
-    
Ecological and individual level analysis of risk factors for HIV infection in four urban populations in sub-Saharan Africa with different levels of HIV infection.

The paper also implies that condoms are not protective against HIV. Again this is not what the preponderance of studies shows:

-    
Condom effectiveness in reducing heterosexual HIV transmission.

The author also notes the reference Allen S et al. (2003) as evidence for the lack of an association between condoms and HIV incidence but the study found that incident HIV infection was found in 5.4% of testing intervals when discordant couples had unprotected sex and in 2.6% of testing intervals when the discordant couples did not have unprotected sex.

**b) The interpretation of the results are not always supported by the data in the paper:**

The Author states:

“ *After adjustments for demographic factors and sexual behavior variables, the measures of knowledge of blood-borne risks become inversely related to HIV infection. That is, respondents who reported that HIV can spread through shared needles/razors and blood contact were less likely to be infected than those who reported that HIV does not spread through such means, once demographics and sexual behaviors were held constant*”.

However, it is unclear how the author reaches this conclusion. The OR for the above relationships are about 0.9 and the confidence intervals are wide. This is not evidence of a relationship.

The author also states:

*“The adjustments for potential confounders also caused the positive associations for knowledge of vaginal sex risk in women and knowledge of anal sex risk in men to disappear. However, the adjusted associations for knowledge of anal sex risk in women and knowledge of vaginal sex risk in men remained meaningfully positive.”*

It is again unclear what “meaningfully positive” means. The OR for women and anal sex has wide CI and doesn’t meet statistical significance. A definition of what the criteria for meaningfully positive are would be useful.

And finally; The OR for men’s understanding of vaginal sex is close to being statistically significant.

**c) The paper’s conclusion is not supported:**

The author claims that:

*“HIV education campaigns in Mozambique and elsewhere in sub-Saharan Africa should include a focus on risks from blood exposures and anal sex.”*

However, the data in the paper doesn’t suggest that this is called for, and even if the data were supportive of the need to do this, there are opportunity costs associated with doing so. What should health workers or health educators not do to create time and money for this change in message?

-     Is the title appropriate for the content of the article? **Yes**
-     Is the abstract a suitable summary of the article? **No (see above)**
-     Is the article well constructed and clear? **No (see above)**
-     Is there adequate analysis, including information on how the data were analyzed (e.g. programs, code, stats etc.)? **There is no information on how the author decided that some results were important**
-     Are the conclusions sensible and balanced? **No (see above).**
-     Have any potential biases or competing interests been disclosed? **Yes**

I have read this submission. I believe that I have an appropriate level of expertise to state that I do not consider it to be of an acceptable scientific standard, for reasons outlined above.

# Response

**Published:** 2012-07-30

**Authors:** Devon Brewer

**Affiliations:** Interdisciplinary Scientific Research, USA

I thank Dr. Livesley for his review. He claims that I cited selectively from the literature and oversimplified issues, and that my interpretation of the results and the conclusions are not supported by the data. In this reply, I demonstrate that these charges are incorrect. Livesley states that “the preponderance of studies show that sexual exposure is by far the most common mode of HIV transmission” and criticizes me for not citing three articles that he thinks support this view. However, there is no solid empirical evidence that vaginal sex is the dominant mode of HIV transmission in sub-Saharan Africa, because studies with designs rigorous enough to determine modes of transmission have not yet been conducted. In the last paragraph of the current article, I wrote “to determine the modes of transmission of HIV in sub-Saharan Africa with confidence, researchers must assess blood and sexual exposures comprehensively in incident HIV cases and controls, trace their contacts corresponding to such exposures, and sequence the DNA in infected persons’ HIV isolates56 57 58.” My colleagues and I have published this plea dozens of times in the last nine years, and no other researchers have attempted to conduct the necessary research. Simply stating that vaginal sex is the dominant mode of transmission, or wishing it to be the case, does not make it so; rather, in science, strong empirical evidence must be presented to support a claim. My colleagues and I carried out a study in Calabar, Nigeria in which we assessed blood and sexual exposures fairly comprehensively in incident HIV cases and controls ( Peters *et al.*, 2009). We found that diverse blood exposures were associated with incident HIV infection. To my knowledge, no epidemiologic study of HIV infection before or since has matched or surpassed the strength of the research design of the Calabar study. Consequently, little can be concluded about the dominant modes of HIV transmission in Africa. Livesley cites Gisselquist D *et al.* (2004), Hauri *et al.* (2004), and White *et al.* (2007) as sources to support the hypothesis that vaginal sex is the dominant mode of HIV transmission in sub-Saharan Africa. These citations that do not provide support for this hypothesis. Gisselquist *et al.* assessed a small set of risk factors (and only one blood exposure, receipt of injections) in relation to incident HIV infection in Rwanda. As I have noted, it is not possible to determine modes of transmission with such a design, especially when blood exposures were so incompletely assessed. Hauri *et al.* and White *et al.*  reported results from modeling exercises on the share of HIV infections that may be due to unhygienic medical injections (just one of many blood exposures encountered by Africans). I and others disagree with many of the assumptions and parameter estimates used and emphasized in their simulations (because they are at odds with empirical data). Their models also lack several key risk components of unhygienic injection practices. I refrain from a detailed technical critique of these studies here. Crucially, neither modeling exercise produced an estimate of the share of HIV transmission due to vaginal sex. Furthermore, modelers have not been able to simulate HIV epidemics that resemble African epidemics with parameter estimates derived from empirical data on Africans’ vaginal sex behavior ( Brewer *et al.*, 2007). Livesley faults me for citing my prior article that showed a strong inverse association between knowledge of blood-borne HIV risks and HIV prevalence at the national level in sub-Saharan Africa ( Brewer, 2011). It is unclear why this is problematic, as the result is robust, and consistent with individual-level results and historical prevention campaign experience. Livesley notes that I did not discuss other possibilities for national variation in HIV prevalence. Such variation is not the focus of my current article; instead, I focused on knowledge of HIV transmission risks in Mozambique. Nonetheless, it is worth evaluating the two sources Livesley cites, both based on the Four Cities Study, for other explanations of variation in HIV prevalence across Africa. The Four Cities Study did not involve assessing participants’ blood exposures. Perhaps even more importantly, the results from the Four Cities Study showed that putative sexual risk factors (such as sex partner concurrency and other high risk sexual behaviors) were inversely associated (in the case of concurrency) or not associated with HIV prevalence in the four cities ( Auvert *et al.*, 2001; Lagarde *et al*. 2001; Rothenberg *et al*., 2002). In my F1000Research article, I stated that there is a “lack of association between condom use in vaginal sex and incident HIV infection14 52 53″ (p. 5) and cited three articles on studies conducted in sub-Saharan Africa (the only three I was aware of at the time that reported on the association or lack thereof). These three articles showed inconsistent results ( Feldblum *et al*., 2010: positive association; Wawer *et al*., 2005: no apparent association; Allen *et al.*, 2003: inverse association), which I summarized as a lack of association. Livesley cites Weller and Davis-Beaty 2007 for the hypothesis that condoms protect against HIV transmission during vaginal sex. However, Weller and Davis-Beaty’s summary included studies from regions outside of Africa, did not account for confounding of condom use with other risk factors (such as common blood exposures in Africa), and did not include Feldblum *et al.* and Wawer *et al.*  results. About half of the studies in Weller and Davis-Beaty’s summary were based on condom use measures that applied to anal sex as well as vaginal sex. Consequently, Weller *et al*. results cannot be used as an estimate of the protective effect of condom use during vaginal sex in sub-Saharan Africa or elsewhere. The protective effect of condom use during anal sex, though, has been well-established in other research. Livesley questions my description of adjusted odds ratios of 0.81 to 0.95 for the association between knowledge of blood-borne HIV risks and prevalent HIV infection as indicating an inverse relationship. By definition, odds ratios under 1.0 reflect inverse or negative relationships. The adjusted odds ratios were in the inverse direction for two different measures of knowledge of blood-borne HIV risks and for both women and men. The confidence intervals for these odds ratios do include 1.0. However, the consistency of the association across measures and sample subgroups, and similarity in magnitude of the association to similar associations in neighboring and nearby countries mean that it is warranted to describe the relationship as inverse. Livesley criticizes me for characterizing the adjusted odds ratios between knowledge of anal sex and HIV infection in women (AOR = 1.18) and knowledge of vaginal sex and HIV infection in men (AOR = 1.85) as “meaningfully positive.” In this context, I intended “meaningfully positive” to refer to associations that were mild to moderate in magnitude. Livesley also correctly noted that “the OR for men’s understanding of vaginal sex is close to being statistically significant.” This result means that Mozambican men who believed that HIV can be transmitted through vaginal sex without a condom were twice as likely to be infected with HIV as men who did not believe vaginal sex was a risk. Livesley argues that the results in my article do not suggest that HIV education programs in sub-Saharan Africa should include a focus on blood-borne risks and anal sex. The results show that Mozambican adults who were unaware of blood-borne transmission were at increased risk for HIV infection, and that children of uninfected mothers who were unaware of blood-borne transmission were at much increased risk for HIV infection. Almost half of Mozambican adults did not know anal sex was an HIV transmission risk. In my opinion, these findings justify including a focus on blood-borne risks and anal sex in HIV education efforts. Livesley further wonders how such topics could be incorporated in HIV prevention and education programming. Successful examples abound. As I discussed in my 2011 article, there is a “heavy emphasis on avoiding blood-borne risks in HIV education programs for primary school students in rich countries [69,70], travelers to poor (especially African) countries [71-73], and employees of the United Nations working in poor countries [74,75]” (p. 194). Most of these educational efforts have been standard and ongoing for over two decades. The risk of anal sex has also been continuously emphasized in HIV prevention and education programs in rich countries. Moreover, during the last 30 years, there have been many instances of HIV education campaigns highlighting blood-borne risks in west and central Africa (Brewer, 2011), although it is uncertain how widespread and consistent these efforts have been. These examples show that people can be more fully informed of the range of HIV risks without additional program cost. The fundamental question is moral and ethical. On what grounds can HIV prevention and education programs in Africa, funded by taxpayers and sponsored by international agencies, foreign aid agencies, and national governments, continue to deprive people of highly relevant, life-saving information?

# Peer review: Referee response for version 1

**Published:** 2012-07-17

**Authors:** Suzanne Gaudreault

**Affiliations:** 1 USAID Health Care Improvement Project, University Research Co., LLC, Bethesda, MD 20814, USA

This is an interesting article, and I think it makes a positive contribution to the evidence base. However, I think the introduction understates the level of knowledge that people with HIV are likely to have about HIV (see below).

The discussion also neglects to consider the fact that due to stigmatization of this disease, many people with HIV do not admit that they have it or even that they have been tested for it.  Unfortunately, this is hard to study or measure, but in practice, I have seen that it is especially true for men, even after they have known about their disease and have been on ART for a long time.  In this study people who admitted to having been tested before were rightfully excluded from the analysis, but the discussion should acknowledge that stigma and other factors may mean that many people who actually knew about their status already (but lied about it) could still have been included in the data, seriously confounding the analysis.

In countries like Mozambique which receive significant foreign assistance to deal with the HIV epidemic  (PEPFAR, Global Fund or other) – people with HIV who are aware of their status typically receive much more intensive education about HIV (through treatment programs and social programs for people with HIV) than the general public.  The content of this education is globally standardized enough that it typically places considerable emphasis on the modes of transmission of HIV. Also, as for people with any health condition, people with HIV are inherently more likely to pay attention to any information on HIV that they come across (public awareness campaigns, etc.).

I have read this submission. I believe that I have an appropriate level of expertise to confirm that it is of an acceptable scientific standard.

# Response

**Published:** 2012-07-19

**Authors:** Devon Brewer

**Affiliations:** Interdisciplinary Scientific Research, USA

Thank you very much, Dr. Gaudreault, for your review.

I agree that it is possible that some previously tested, infected persons might deny having tested previously and that they might have learned about blood exposures and anal sex as routes of transmission during HIV testing or clinical care. However, several lines of evidence suggest that such “knowledge through denied prior testing” is rare, and that even if this bias were present, it cannot account for the association between knowledge and HIV infection.

In Class’ (2012) research, adult HIV patients and parents and other caretakers of HIV infected children in Maputo, Mozambique, responded to open-ended questions about routes of HIV transmission. Fifty-five percent (12/22) of the adult HIV patients and 62% (16/26) of parents and caretakers were aware of blood-borne modes of HIV transmission. All respondents aware of blood-borne HIV transmission reported learning of these modes of transmission exclusively from unofficial, non-clinical, non-public health sources, such as “on the street.” Class’ (2012) respondents reported that none of their clinical providers ever discussed blood-borne transmission as a potential explanation for how infection occurred.

It is unclear the extent to which blood-borne HIV risks are covered in HIV counseling in southern Africa. As I demonstrated in this article and a prior report (reference 18), public HIV education campaigns in southern Africa have not in the past and do not currently address blood-borne HIV risks, even though they are often funded and guided by large international aid agencies such as USAID, the Global Fund, PEPFAR, DFID, and UNAIDS. Therefore, it is reasonable to question how well such risks are addressed in these countries’ HIV counseling programs.

Any potential bias in reporting prior HIV testing by infected persons cannot account for the strong national-level inverse association between knowledge of blood-borne HIV risks and HIV infection (see reference 18). Countries in which many people are aware of such risks have much lower HIV prevalences than countries in which few people are aware of these risks.

Also, potential bias in reporting prior HIV testing by infected persons is irrelevant to the association between uninfected mothers’ knowledge of blood-borne HIV risks and HIV infection in their children (reported in the current article). Children of uninfected mothers who know about these risks are much less likely to be infected than children of uninfected mothers who do not know about these risks.

Furthermore, even if infected persons who denied prior HIV testing were included in the data I analyzed, their inclusion could not generate the observed inverse association between knowledge of blood-borne risks and HIV infection. If they had learned about blood-borne risks during testing or clinical care, and reported such awareness in the Mozambique AIS, their responses would produce a tendency toward a positive, rather than inverse, association between knowledge of blood-borne risks and HIV infection.

Reference

Class D. The socio-political economy of antiretroviral treatment as HIV prevention . Doctoral dissertation, University of Trento, 2012. Available at http://eprints-phd.biblio.unitn.it/803/.
